# Analysis of risk factors in patients with alcohol delirium who have been treated at the riga psychiatry and narcology center in 2018

**DOI:** 10.1192/j.eurpsy.2021.2165

**Published:** 2021-08-13

**Authors:** K. Kurzemnieks

**Affiliations:** Narcology, Riga Psychiatry and Narcology Center, Rīga, Latvia

**Keywords:** delirium tremens, Predictors, alcoholism, alcohol withdrawal

## Abstract

**Introduction:**

Alcohol abuse can be the cause for psychotic disorders. In the International Classification of Diseases (ICD10) they are coded F10.4-F10.9. One of the potentially life-threatening complications is the development of alcohol delirium. Mortality rates in patients with untreated alcohol delirium reach 15%. It is extremely important to identify the risk factors that contribute to the development of delirium in time to ensure the most effective treatment and to ensure the patient’s potential survival in the hospitalization and post-hospitalization phase.

**Objectives:**

To analyze and evaluate the risk factors that have coused alcohol withdrawal with the development of delirium in patients admitted at the department of Narcology of the Riga Psychiatry and Narcology Center in 2018.

**Methods:**

This study is a retrospectively conducted cohort study based on data from inpatient medical records for patients diagnosed with alcohol-induced delirium at the Department of Narcology of the Riga Psychiatry and Narcology Center in Year 2018.

**Results:**

In the Riga Psychiatry and Narcology Center 113 patients were diagnosed alcohol caused delirium. That makes up to 8% of all inpatients in year 2018. Summary of the prevalence of the most significant risk factors in 2018 inpatients with alcohol delirium.

**Table:** 

High levels of aspartate aminotransferase	95%
Tachycardia	76%
High levels of alanine aminotransferase	54%
Low platelet count	51%
High systolic blood pressure	50%
High diastolic blood pressure	46%
Other somatic diseases	45%
Previous history of detoxification	37%
History of alcohol-induced seizures	13%

**Conclusions:**

The study indicated that some easily determined parameters are potential clinical predictors for the development of delirium tremens.

**Disclosure:**

No significant relationships.

